# Synthetic cooling agents enhance nicotine reinforcement and mesolimbic dopamine signaling

**DOI:** 10.1038/s41386-026-02485-0

**Published:** 2026-07-23

**Authors:** Sarah K. Maddox, Esther N. Mensah, Lauren E. Young, Hannah D. Strcula, Shane V. Perelman, M. Kyle Sword, Brandon J. Henderson

**Affiliations:** Department of Biomedical Sciences, Joan C Edwards School of Medicine at Marshall University, Huntington, WV USA

**Keywords:** Addiction, Neurophysiology

## Abstract

Flavor additives in electronic delivery systems (ENDS) have been shown to enhance nicotine reinforcement. Although some states and cities have pursued implementation of menthol bans, synthetic coolants such as WS-3 and WS-23 have emerged as substitutes. In this study, we investigated the impact of WS-3 and WS-23 on nicotine self-administration behavior and dopamine (DA) release in the nucleus accumbens (NAc) core. To model nicotine reinforcement, we used an E-Vape® self-administration (EVSA) assay and observed that male and female mice exposed to nicotine plus WS-3 and WS-23 exhibited enhanced self-administration behavior when compared to mice exposed to nicotine alone or PGVG (control treatment). We also observed that the enhancement in nicotine self-administration is similar to what is observed with menthol. To measure changes in dopamine release, we used fast-scan cyclic voltammetry (FSCV) and fiber photometry. Here, we observed that WS-3 and WS-23 increased the amount of dopamine released in the NAc under both tonic (5 Hz) and phasic (60 Hz) stimulation conditions when compared with nicotine-alone. Fiber photometry recordings showed that the WS-3 and WS-23 enhanced nicotine-stimulated dopamine release in the NAc core. Using electrophysiology and microscopy assays, we observed that synthetic coolants enhance the intrinsic excitability of ventral tegmental area (VTA) dopamine neurons and enhance nicotine-induced upregulation of nicotinic acetylcholine receptors (nAChRs). In both FSCV and photometry assays, synthetic coolants produced enhancements in dopamine signaling that were comparable in magnitude to those observed with menthol. These findings demonstrate that synthetic cooling agents enhance nicotine reinforcement and are associated with increased mesolimbic dopamine signaling, enhanced VTA dopamine neuron excitability, and increased nicotine-induced nAChR upregulation.

## Introduction

Menthol is a common additive in cigarettes and e-cigarettes, known for its cooling sensation and its role in enhancing nicotine’s addictive properties [1–3]. In recent years, nicotine products have shifted in response to states regulating the use of menthol in nicotine products due to its association with increased nicotine abuse liability. This has resulted in manufacturers substituting them with synthetic coolants, like WS-3 and WS-23 [4–7].

WS-3 and WS-23 are flavorless compounds similar to menthol because they provide the same cooling effect while lacking menthol’s strong mint flavor [6]. These products are currently regulated in food products but not for inhalation. We have previously shown that menthol enhances nicotine’s addictive properties by altering nicotinic acetylcholine receptor (nAChR) activity and increasing dopamine (DA) release in the nucleus accumbens (NAc) core, which is a key brain region involved in reward and motivation [8–10]. Menthol’s ability to enhance nicotine reinforcement has been linked to its ability to enhance nicotine-induced upregulation of nAChRs [10, 11] and dopamine neuron excitability [10], and nicotine-induced changes in dopamine release [8, 12]. This promotes enhancements in nicotine self-administration [9, 13–15]. WS-3 and WS-23 were proposed as menthol replacements in topical shaving cream products, and due to this, many WS-series compounds exhibit functional and structural characteristics of menthol [6]. Studies have investigated the inclusion of WS-3 and WS-23 in tobacco and vape products and showed that products contained WS-3 and WS-23 in combination at concentrations up to 36 mg/mL [7, 16, 17]. Additionally, WS-3 and WS-23 exhibit similar pharmacological activity at TRPM8 receptors (EC_50_ values of 3.7 and 44 µM, respectively) as menthol (EC_50_ of 4 µM) [18–20]. Menthol can act as an allosteric agent on nAChRs at high concentrations [21–23] and a chemical chaperone of nAChRs at low concentrations [10, 24, 25]. Thus, we hypothesized that the combination of WS-3 and WS-23 would promote changes in nicotine reinforcement like menthol. Hence, we investigated how synthetic coolants impact the addictive properties of nicotine.

To accomplish this, we employed an e-Vape® self-administration (EVSA) model, which we previously used to determine the impact of chemical flavors on addiction-related behaviors [9, 13, 26–30]. We assigned mice to e-liquid conditions in a between-subjects design to determine how nicotine plus synthetic coolants or menthol impacted reinforcement-related behaviors. We also used Fast-scan cyclic voltammetry (FSCV) and fiber photometry to assess whether synthetic coolants alter NAc core dopamine release. We observed that the combination of WS-3 and WS-23 enhanced nicotine self-administration. Furthermore, we observed synthetic coolants enhanced nicotine-induced changes in dopamine release, nAChR upregulation, and self-administration similar to menthol.

The results of this study provide insight into how synthetic coolants may contribute to nicotine reinforcement. Understanding these effects will help inform and shape future regulatory decisions and public health strategies related to the inclusion of synthetic coolants in nicotine products.

## Material and methods

### Mice

Experiments were conducted following the National Institutes of Health guidelines for care and use of animals and approved by the Marshall University IACUC. Mice were group housed on a standard 12/12-h light/dark cycle at 22^o^C and given food and water ad libitum. Mice were young adults (10 weeks old), male and female C57BL/6 J, at the time of starting viral injections. For electrophysiology assays, male and female α6-GFP mice were used. For microscopy, male and female α4-mCherryα6-GFP mice were used.

### Drugs and E-liquid composition

Nicotine ditartrate dihydrate (Acros Organics, AC415660500), (-)-menthol (Alfa Aesar, A10474), WS-3 (TCI, E0796), and WS-23 (TCI, I0729) were dissolved in Propylene glycol (Tedia, PR1494-065) and vegetable glycerin (J.T. Baker, 2143-01) (PGVG; 50:50 ratio). Menthol had a final concentration of 15 mg/mL while nicotine was 6 mg/mL. E-liquids containing synthetic coolants were 7.5 mg/mL WS-3 and 7.5 mg/mL WS-23 (15 mg/mL total coolant concentration). 6 mg/mL nicotine produces the highest earned responses in male mice [9] and flavor-induced enhancement with menthol and green apple in both male and female mice [13]. 6 mg/mL nicotine is common in tank-based vape devices [31]. Similarly, total flavor concentration for e-liquids is ~15 mg/mL [31]. Thus, we designed the total flavor concentrations for menthol and coolants to this concentration, similar to our previous flavor studies [9, 13, 27, 28, 30].

### E-Vape® Self-administration (EVSA) assays

We used a commercial setup (LJARI, La Jolla, CA, USA; www.ljari.tech) [9, 29]. Two standard Med-Associates nose pokes (containing yellow cue lights) were mounted on the walls of the chamber in a dark Plexiglas enclosure that minimized extraneous light and noise. Airflow was vacuum controlled by an electric pump (1 L/min). Geek Vape® tanks (0.40Ω dual coil) were activated by a custom e-cigarette mod box (LJARI, La Jolla, CA, USA, 400 °F, 65 W).

Male and female mice began EVSA on a fixed-ratio 1 (FR1) self-administration schedule on a Monday for 5 daily 2-h sessions, with a weekend abstinence period. Mice were singly placed into air-tight operant chambers. Active nose pokes resulted in a 3 s delivery of vaporized e-liquids through the vapor port with a 30 s timeout. Vapor is eliminated from the chamber in <20 s. During the timeout, a yellow cue-light remained on in the active nose poke hole. Inactive nose pokes were recorded with no consequences. After session 5, mice were transitioned to a FR2 schedule for 5 sessions and then FR3 for 5 sessions. Self-administration was conducted in the light phase of the light cycle.

### Photometry

Under isoflurane anesthesia, 700 nL of AAV9-CAG-dLight1.3b (Addgene) was injected (100 nL/min) through a syringe (Hammilton) into the NAc core (AP: +1.2, ML: 1.2, DV: 5.2) of 10-week-old male and female mice (*n* = 4-5 males, 4-6 females). In the same surgery, a fiber optic cannula (5 mm in length) was inserted and cemented in place (Metabond). Data were collected 4 weeks later to facilitate peak virus expression. For recording, excitation (470 nm) and isobestic (415 nM) LED channels were cycled in 10 ms frames (Neurophotometrics, FP3002). Timestamps for cue and eVape deliveries were extracted to csv files. Functional fluorescence signals (ΔF/F and Z-scores) were analyzed using a guided photometry analysis suite (GuPPy) developed by the Lerner lab [32]. Timestamps (TTLs) for cue (light off and solenoid click) and eVape delivery (when vape tank was triggered; 1^st^ poke on FR1, 2^nd^ poke on FR2, and 3^rd^ poke on FR3) were used to align specific events in GuPPy.

### Passive vapor exposure

**Fig. 1:**
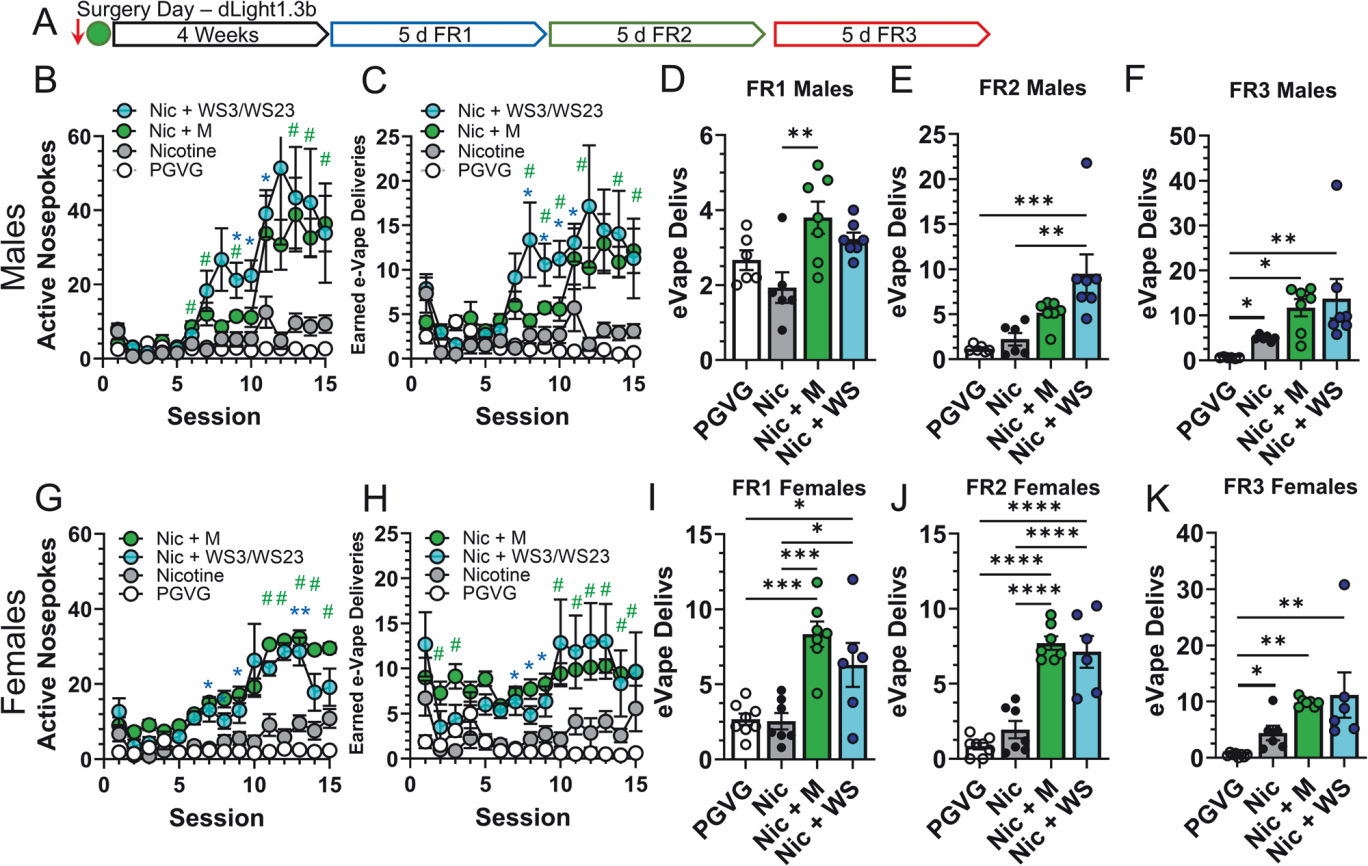
Synthetic coolants enhance nicotine vapor self-administration. **A** Experimental timeline. **B, G** Active nose poke and earned eVape delivery **C, H** comparison for male and female mice assigned to PGVG, nicotine-only, nicotine plus menthol, and nicotine plus WS-3/WS-23. Mean eVape deliveries for FR1 **D, I**, FR2 **E, J**, and FR3 **F, K**. Data are mean ± SEM. For each e-liquid assignment, *n* = 6-7 mice. In **B, C, G** and **H**, data were analyzed by repeated measures two-way ANOVA, (#, indicates difference between PGVG and nicotine plus menthol and * indicates difference between nicotine plus PGVG and W-S3/WS-23). In **D–F** and **I–K**, data were analyzed by one-way ANOVA. *, *p* < 0.05; **, *p* < 0.01; ***, *p* < 0.005, *post hoc* Bonferroni (see Tables S1–S5).

Additional cohorts of mice were exposed passively to PGVG, nicotine, or nicotine plus coolants for 10 daily 2-h sessions at a rate of 15 deliveries per session (3 s puff). We chose this value since both male and female mice exhibited an average of 15 earned eVape deliveries during the FR3 period (see Fig. 1). Later, mice were euthanized and brains were extracted for patch-clamp electrophysiology and FSCV (*n* = 4–6 males, 4–6 females, each group) or confocal microscopy (*n* = 4–5 males, 4–6 females).

#### Patch-clamp electrophysiology and fast-scan cyclic voltammetry

Mice were exposed to CO_2_, then cardiac perfused with ice-cold NMDG-based artificial cerebrospinal fluid (NMDG-ACSF) saturated with 95%/5% O_2_/CO_2_ (carbogen) containing (in mM): 93 NMDG, 2.5 KCl, 1.2 NaH_2_PO_4_, 10 MgSO_4_, 0.4 CaCl_2_, 30 NaHCO_3_, 5 Na-ascorbate, 3 Na-pyruvate, 2 thiourea, and 25 glucose. Brains were placed in agarose for slicing (Compresstome^®^ VF-300-OZ, Precisionary Instruments). Coronal brain sections (300 µm) were cut into cold carbogenated NMDG-ACSF to obtain slices containing the VTA (bregma -2.8 to -3.4 mm) or NAc core (for FSCV). Slices recovered at 32°C in carbogenated NMDG-ACSF for 12-15 min. Next, slices were transferred to standard ACSF containing (mM): 125 NaCl, 2.5 KCl, 1.2 NaH_2_PO_4_, 1.2 MgCl_2_, 2.4 CaCl_2_, 26 NaHCO_3_, and 11 glucose for one hour at 32°C. One hour later, slices were transferred to the recording chamber and perfused with carbogenated ACSF (1.5–2.0 ml/min) at room temperature.

Neurons were visualized with an Axio Examiner A1 (Zeiss) with an Axiocam 702 mono. We identified putative VTA dopamine neurons using α6-GFP. We recorded electrophysiological signals with an Integrated Patch-Clamp Amplifier (Sutter) using previously described methods [33–35]. Electrodes had resistances of 4–10 MΩ when filled with intrapipette solution (in mM): 135 K gluconate, 5 KCl, 5 EGTA, 0.5 CaCl_2_, 10 HEPES, 2 Mg-ATP, and 0.1 GTP. Recordings were sampled at ≥10 KHz. Series resistance was monitored without compensation and recordings were terminated if series resistance changed by >20%. In whole-cell recordings, recordings were made after 5 min to provide sufficient time for interchange of intrapipette solution with intracellular components.

Intrinsic excitability was assessed by using a stepped current injection protocol in current clamp mode (5 pA steps, -20 pA to 90 pA). Rheobase was determined from the threshold current sufficient to elicit the first action potential. Electrophysiological data (Rheobase) was correlated to the mean FR3 active nosepokes from all 5 of the individual mouse’s FR3 sessions (FR3 score, sessions 11 – 15). For each mouse, we collected data from 3-4 VTA dopamine or GABA cells and calculated the average rheobase and firing frequency values.

For FSCV, slices were transferred to the recording chamber and perfused with carbogenated ACSF (1.5–2.0 ml/min) at room temperature. Recording and stim electrodes were placed in the NAc using a 4X or 10X objective. Tonic (5 pulses, 5 Hz) or phasic (5 pulses, 60 Hz) electrical stimulation (350 µA) was triggered with a Master-9 stimulator (AMPI), driven by Demon Voltammetry software. Voltage was swept from −0.4 to 1.2 mV (20 Hz) using a VA-10X voltammeter (NPI electronic GmbH) and controlled with Demon Voltammetry software. Commercial FSCV electrodes were used (CFE-2, NPI electronic GmbH) to interface with the VA-10 headstage. FSCV electrodes were calibrated using 0.1, 1, 10 µM dopamine standard solutions.

### Confocal microscopy

α4-mCherryα6-GFP mice were culled, and brains were extracted and quickly frozen on a slurry of dry ice and acetone. Brains were frozen for ≥24 h and then sectioned at 20 μm using a cryostat (Leica CM1950). We collected brain sections containing the VTA (target bregma, -3.1; AP limits, -3.4 → -2.9). VTA cells were imaged using an Olympus FV3000. Calculations of raw integrated density (RID) were completed using previously established methods [28, 36]. As conducted previously, density of nAChRs containing both an α4 and α6 nAChR subunit in pentameric assembly (α4α6* nAChRs) was determined using pixel-based (FRET (NFRET) methods) [28, 36].

### Statistics

Experimenters for electrophysiology and FSCV assays were blind to experimental conditions. Data were analyzed with one-way or two-way ANOVAs depending on comparisons using GraphPad Prism. For electrophysiological analyses, the mouse was considered the experimental unit for rheobase and maximum firing frequency measurements. Current-voltage plots were analyzed using a mixed-effects model with current as a repeated factor and neuron nested within mouse. F stats are provided in the *Results* section, and complete statistics are provided in *Supplementary Material* (Tables S1–S12).

## Results

### Synthetic coolants enhance nicotine self-administration behavior

To determine how synthetic coolants impact nicotine reinforcement, we employed an EVSA assay using adult male and female mice. The self-administration behavior of mice was studied across four groups (PGVG, nicotine alone, nicotine plus menthol, or nicotine plus WS-3/WS-23). To assess reinforcement, mice completed five sessions each at fixed-ratio 1 (FR1), FR2, and FR3 schedules over the course of three weeks (Fig. 1A).

Over the EVSA paradigm, mice assigned to nicotine-alone, nicotine plus menthol, and nicotine plus coolants increased their active nose poke responses (Fig. 1B, G, Fig. S1; effect of e-liquid, F_(3,22)_ = 6.7, *p* = 0.0022 and F_(3,24)_ = 76.7, *p* < 0.0001 for males and females, respectively) and earned eVape deliveries (Fig. 1C, H; effect of e-liquid, F_(3,22)_ = 7.4, *p* = 0.0014 and F_(3,24)_ = 28.2, *p* < 0.0001 for males and females, respectively) during the FR1, FR2, and FR3 sessions. During FR1 sessions, male mice assigned to nicotine plus menthol earned more eVape deliveries (rewards) than nicotine-alone (Fig. 1C; main effect, F_(3,22)_ = 5.61, *p* = 0.0052) while females earned more with nicotine plus menthol and nicotine plus WS-3/WS-23 compared to PGVG (control) (Fig. 1I; main effect, F_(3,24)_ = 12.02, *p* < 0.0001). Mice assigned to nicotine plus WS3/WS23 earned more eVape deliveries compared to PGVG and nicotine-only during the FR2 phase (Fig. 1E, J; main effect F_(3,22)_ = 9.212, *p* = 0.0004 and F_(3,24)_ = 35.66, *p* < 0.0001, males and females, respectively). During FR3 sessions, mice assigned to nicotine plus menthol and nicotine plus WS3/WS23 earned more eVape deliveries than PGVG (Fig. 1F, K; main effect F_(3,22)_ = 5.40, *p* = 0.006 and F_(3,24)_ = 7.34, *p* = 0.0013, males and females, respectively).

### Synthetic coolants increase NAc dopamine release

In a subset of the mice assigned to our EVSA paradigm, dLight1.3b was injected into the NAc core. During FR3 sessions 12 and 14, cue-stimulated and eVape-stimulated fluorescence was monitored for the first hour of each EVSA session for mice assigned to PGVG, nicotine, nicotine plus menthol, and nicotine plus WS-3/WS-23 (main effect F_(3,31)_ = 10.16, *p* < 0.0001 and F_(3,31)_ = 47.6, *p* < 0.0001, cue-stimulated and eVape-stimulated, respectively).

**Fig. 2:**
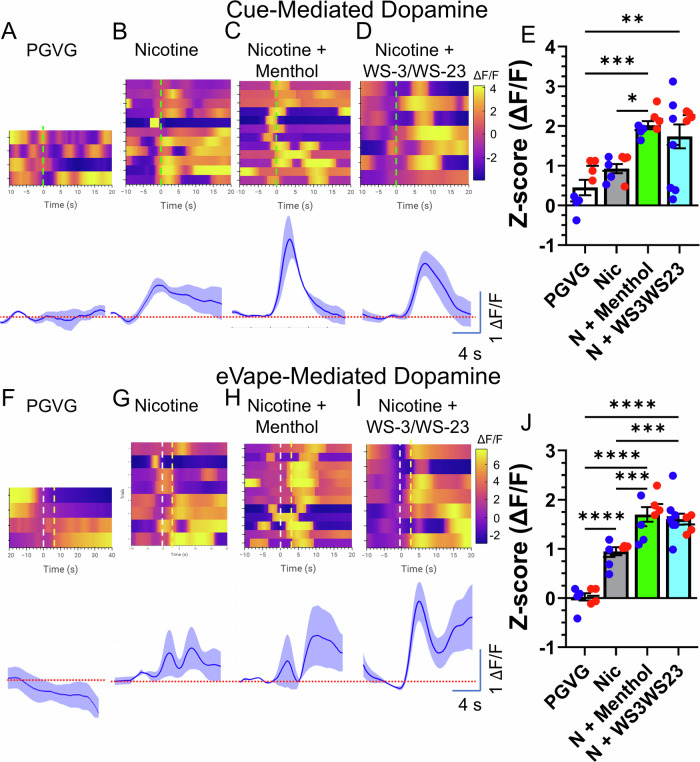
Synthetic coolants enhance cue-stimulated and reward-stimulated dopamine in the NAc core. (**A–D**, top) Representative fiber photometry heat maps for cue-stimulated dLight1.3b fluorescence for mice exposed to PGVG, nicotine-alone, nicotine plus menthol, and nicotine plus WS3/WS23. (**A–D**, bottom) Representative cue-stimulated dLight1.3b mean fluorescent waveforms for individual sessions for mice exposed to PGVG, nicotine-alone, nicotine plus menthol, and nicotine plus WS3/WS23. **E** Mean ΔF/F for cue-stimulated dLight1.3b fluorescence in the NAc core. (**F, I**, top) Representative fiber photometry heat maps for eVape-stimulated dLight1.3b fluorescence for mice exposed to PGVG, nicotine-alone, nicotine plus menthol, and nicotine plus WS3/WS23. (**F–I**, bottom) Representative dLight1.3b eVape-stimulated mean fluorescent waveforms for individual sessions for mice exposed to PGVG, nicotine-alone, nicotine plus menthol, and nicotine plus WS3/WS23. **J** Mean ΔF/F for eVape-stimulated dLight1.3b fluorescence in the NAc core. Individual dots represent mean values for individual mice (*n* = 6–11 mice, males [blue] and females [red] combined). Data are mean ± SEM. Data were analyzed by one-way ANOVA (Table S6). In heatmaps, each is a representative 2 h EVSA session for an individual mouse where each row represents an independent trial (cue-delivery or eVape delivery).

For cue-mediated dopamine release, PGVG-assigned mice exhibited a mean ΔF/F of 0.45 ± 0.19, mice assigned to nicotine-only mice generated a mean ΔF/F of 0.93 ± 0.11, mice assigned to nicotine plus menthol demonstrated a mean ΔF/F of 2.02 ± 0.10, and mice assigned to nicotine plus WS3/WS23 mice produced a mean ΔF/F of 1.74 ± 0.11 (Fig. 2A–E). Nicotine plus menthol and nicotine plus WS3/WS23 were significantly different to PGVG (*p* < 0.05, Fig. 2E).

For eVape-stimulated dopamine release, PGVG-assigned mice yielded a mean ΔF/F of 0.025 ± 0.07, mice assigned to nicotine-only mice displayed a mean ΔF/F of 0.95 ± 0.08, mice assigned to nicotine plus menthol mice exhibited a mean ΔF/F of 1.7 ± 0.15, and mice assigned to nicotine plus WS3/WS23 mice revealed a mean ΔF/F of 1.6 ± 0.11 (Fig. 2F-J). Nicotine (*p* < 0.001), nicotine plus menthol (*p* < 0.001), and nicotine plus WS3/WS23 (*p* < 0.001) were significantly different from PGVG (Fig. 2J). Both nicotine plus menthol and nicotine plus WS3/WS23 were significantly different from nicotine-alone (*p* < 0.005, Fig. 2J).

The fluorescent signatures of dLight1.3b signals were distinct between cue-stimulated and e-vape-stimulated signatures (Fig. 2). Cue-stimulation produced a more rapid rise time in fluorescence (Fig. 2A–D) while e-Vape-stimulated fluorescence exhibited a slower rise-time. Likely due to the differences in the event times, as the cue is a distinct event (light plus click), while the triggered vapor is delivered over a three-second period and is dependent upon inhalation.

**Fig. 3:**
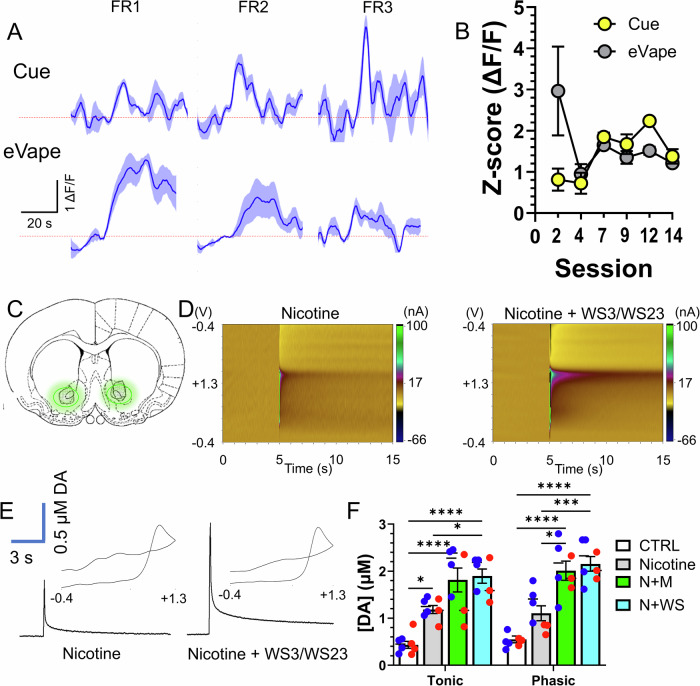
Synthetic coolants enhance electrically-evoked NAc core dopamine release. **A** Representative NAc dLight1.3b signal from cue-stimulated and eVape-stimulated events during FR1, FR2, and FR3 sessions. **B** Mean Z-score (ΔF/F) for NAc dopamine release for FR1 (session 2, 4), FR2 (session 7, 9), and FR3 (session 12, 14). **C** Schematic showing the target location of recording electrode placement in the NAc core. **D** FSCV color plots showing dopamine release in NAc core from a phasic (5p, 60 Hz) stimulation in mice assigned to nicotine-alone or nicotine plus WS-3/WS-23. **E** Representative FSCV waveforms and voltammograms (inset) for phasic stimulation in nicotine-treated and nicotine plus WS3/WS23-treated brain slices. **F** Mean, calibrated dopamine release in response to tonic (5p, 5 Hz) and phasic (5p, 60 Hz) stimulation across the four treatment groups. Individual dots represent mean data for individual mice (*n* = 7-9 mice, males [blue] and females [red] combined). Data are mean ± SEM. FSCV was analyzed by two-way ANOVA (Table S7).

For mice assigned to nicotine plus WS3/WS23, we further analyzed photometry signals captured throughout the paradigm (FR1, sessions 2 and 4; FR2, sessions 7 and 9; FR3, sessions 12 and 14) (Fig. 3A, B). We observed that eVape-stimulated dopamine release was highest earlier in the paradigm (FR1), but cue-stimulated dopamine release increased as the paradigm progressed (Fig. 3B).

FSCV was also performed in brain slices containing the NAc core. For these experiments, mice were non-contingently exposed to vaporized e-liquids for 10 2-h sessions. Each session included 15 vapor deliveries (3 s puff, same as EVSA), and mice were assigned to one of four groups: PGVG, nicotine alone, nicotine plus menthol, or nicotine plus WS-3/WS-23. Following the final vapor exposure, brain slices containing the NAc were collected and electrically stimulated to evoke dopamine release. We examined tonic (5 pulses, 5 Hz) and phasic (5 pulses, 60 Hz) stimulation to investigate how synthetic coolants affected dopamine release compared to the other groups (main effect of e-liquid F_(3,50)_ = 46.02, *p* < 0.0001).

Under tonic stimulation, both the nicotine plus menthol and nicotine plus WS3/WS23 groups were shown to have significantly more dopamine release than the PGVG group (*p* < 0.0001, Fig. 3F). The nicotine plus WS3/WS23 group released significantly more dopamine than the nicotine-alone group (*p* < 0.05). This suggests that WS-3 and WS-23 enhance dopamine release more than nicotine alone. Under phasic stimulation, the difference in dopamine release between the groups was more pronounced. The synthetic coolant group had significantly more dopamine release than the nicotine-alone (*p* < 0.001) or PGVG group (*p* < 0.0001). The menthol group also saw increased dopamine release under phasic stimulation, but to a lesser extent than the synthetic coolant group. These findings show that WS-3 and WS-23 enhance dopamine release in the NAc to a greater magnitude than nicotine alone. The increase in dopamine release is important because dopamine is one of the main chemicals involved in reinforcement and reward. This data suggests that synthetic coolants increase dopamine release beyond what nicotine alone does, which would reflect the enhanced behavioral responses we saw in the EVSA assays.

We assigned a separate cohort of mice to WS-3/WS-23-alone (zero-nicotine) and observed that male and female mice did not discriminate between the active and inactive nose pokes during the EVSA paradigm (Fig. S2, active:inactive ratio <2). Additionally, we observed that while mice assigned to coolants-only exhibited cue-stimulated dopamine release (ΔF/F, 1-1.5), they did not exhibit robust eVape-stimulated dopamine release (ΔF/F ≤ 0.5, Fig. S2).

#### Synthetic coolants enhance VTA dopamine neuron intrinsic excitability but not nicotine-stimulated neuronal excitability

**Fig. 4:**
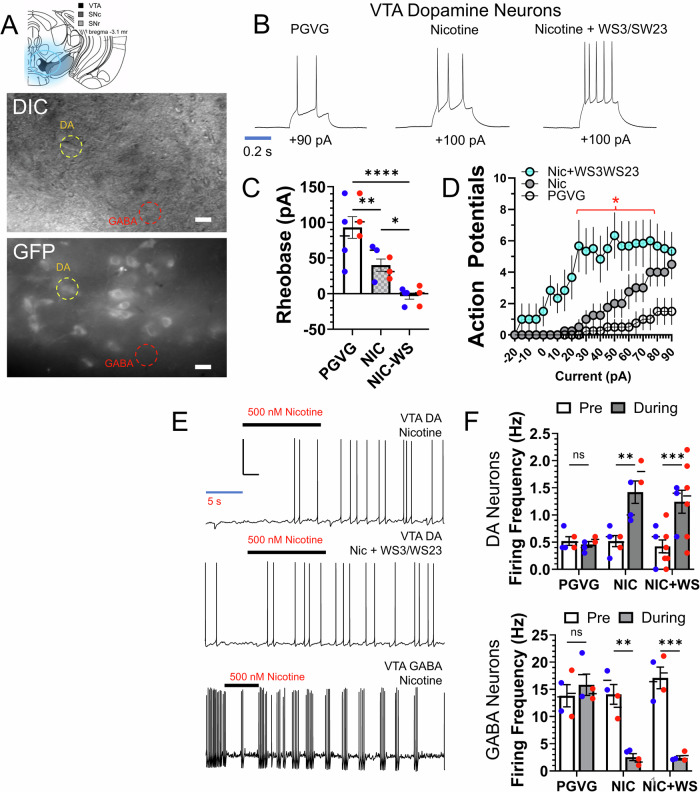
Synthetic coolants enhance intrinsic excitability of VTA dopamine neurons. **A** Schematic and representative DIC and GFP images of putative VTA a dopamine neuron (bars, 20 µm). **B** Representative images of current-clamp waveforms used to determine maximum step frequency. **C** Mean rheobase and maximum step frequency. Individual dots represent mean values from individual mice. **D** Mean current-voltage (IV) plot for VTA dopamine neurons. **E** Representative current-clamp recordings of VTA dopamine (top two) or VTA GABA (bottom) neurons responding to acute applications of 500 nM nicotine. **F** Mean firing frequency prior to and during nicotine applications for VTA dopamine and GABA neurons. Data are mean ± SEM. Individual dots are individual mice (blue, males; red, females) and data were analyzed by one-way ANOVA **C** or two-way ANOVA **D, E** (Tables S8–S11).

We previously showed that menthol enhances nicotine-induced changes in the excitability of VTA dopamine neurons [10]. This is because menthol facilitates the stability and upregulation of high sensitivity α4α6* nAChRs on the VTA [10]. To determine if synthetic coolants alter the excitability of VTA dopamine neurons, we used a new cohort of α6-GFP mice to examine changes in the excitability of VTA dopamine neurons. Mice were non-contingently exposed to PGVG, nicotine, or nicotine plus WS3/WS23 (15 eVape deliveries per 2 h sessions, 10 sessions). Following the completion of the non-contingent paradigm, acute brain slices were prepared for patch-clamp electrophysiology. The presence of α6-GFP was used to identify putative VTA dopamine neurons (α6-GFP has >95% overlap with tyrosine hydroxylase [34, 37]). To determine intrinsic excitability, a current-step protocol was used to determine rheobase (minimum current needed to trigger an action potential) and the maximum firing frequency during current steps (Fig. 4).

Based on a one-way ANOVA, there was a significant effect of e-liquid assignment on rheobase (main effect F_(2,17)_ = 21.6, *p* < 0.0001). PGVG-mice exhibited a mean rheobase of 92.9 ± 15.2 pA (Fig. 4C). As expected, nicotine enhanced the excitability of VTA dopamine neurons. We observed that nicotine-exposed mice exhibited a mean rheobase of 40.0 ± 8.7 pA, a significant enhancement in excitability compared to PGVG-exposed mice (*p* = 0.010). Mice exposed to nicotine plus coolants had a mean rheobase of -15.8 ± 3.3 pA, which is a significant enhancement compared to nicotine-alone (*p* = 0.0092) and PGVG (*p* < 0.0001). Based on a one-way ANOVA, there was also a significant effect of e-liquid assignment on firing frequency (Fig. 4C, Table S8). Analysis of the current-voltage plot showed a significant effect of e-liquid assignment (Table S8). Mice exposed to nicotine plus WS3/WS23 produced a significant increase in the number of action potentials for current steps (Fig. 4D, Table S9).

Next, we examined how VTA dopamine neurons responded to acute applications of nicotine (500 nM, 10 s local application) (Fig. 4E; e-liquid, F_(2,16)_ = 4.08, *p* = 0.037; acute nicotine, F_(1,16)_ = 31.4, *p* < 0.0001; interaction, F_(2,16)_ = 10.6, *p* = 0.0012). In PGVG-exposed mice, 500 nM nicotine did not produce a significant difference in firing frequency of VTA dopamine neurons (Fig. 4F). VTA dopamine neurons in Nicotine-exposed animals exhibited a ~ 3-fold increase in firing frequency during the acute nicotine application (*p* < 0.01, Fig. 4F). VTA dopamine neurons prepared from mice exposed to nicotine + WS3/WS23, we also observed a ~ 3-fold increase (*p* < 0.001) following acute nicotine exposure similar to nicotine-alone (Fig. 4F). Several studies have identified that VTA GABA neurons become susceptible to desensitization following long-term exposure to nicotine. Similarly, we applied acute nicotine to current-clamped VTA GABA neurons (e-liquid, F_(2,9)_ = 7.69, *p* = 0.011; acute nicotine, F_(1,9)_ = 44.4, *p* < 0.0001; interaction, F_(2,9)_ = 17.9, *p* = 0.0007). Like our prior observation with VTA dopamine neurons, VTA GABA neurons from PGVG-exposed mice demonstrated no significant change in firing frequency during acute application of 500 nM nicotine (Fig. 4F). For VTA GABA neurons from nicotine-exposed and nicotine + WS3/WS23, we exhibited a nearly identical reduction in firing frequency during acute nicotine exposure (Fig. 4F).

#### Synthetic coolants increase nicotine-induced upregulation of nAChRs on VTA Dopamine Neurons

**Fig. 5:**
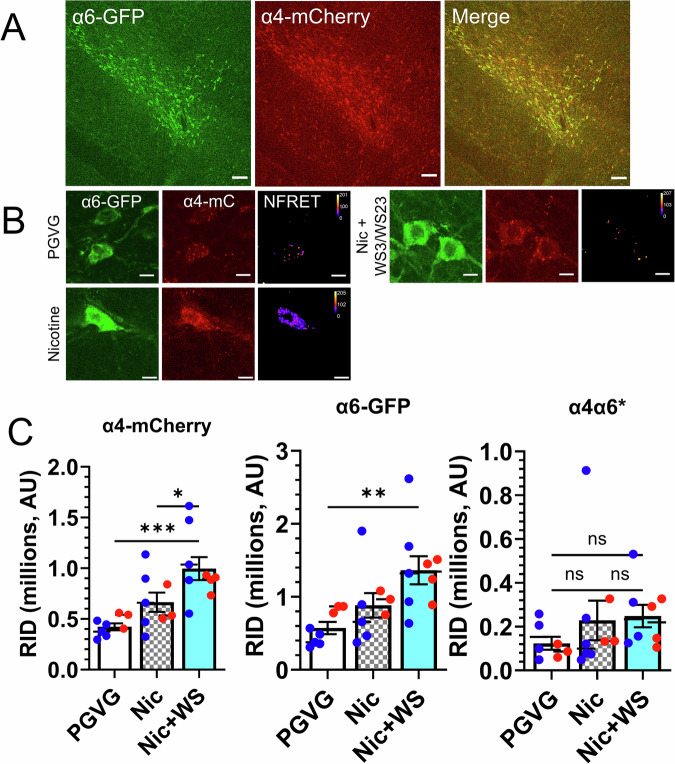
Synthetic coolants enhance nicotine-inducted upregulation of nAChRs. **A, B** Representative 10X **A** and 20X (**B**, with 5X digital zoom) images from a α4-mCherryα6-GFP mice. **C** Mean RID of α4-mCherry, α6-GFP and α4α6* fluorescence in VTA dopamine neurons. In **C**, data are mean ± SEM. *, *p* < 0.05; **, *p* < 0.01, one-way ANOVA with *post hoc* Tukey (Table S12). Scale bars are 100 µm (10X images) or 10 µm (20X, 10X digital zoom images). Blue dots, males; red dots, females.

Nicotine-induced upregulation of nAChRs has been associated with long-term smoking for many decades [38–42]. We have shown that nicotine-induced upregulation correlates with reward-related behavior [34] and reinforcement-related behavior [26–28]. Thus, we used α4-mCherryα6-GFP mice to examine changes in nAChR density in VTA dopamine neurons following exposure to synthetic coolants (Fig. 5; α4-mCherry, F_(2,22)_ = 10.5, *p* = 0.0006; α6-GFP, F_(2,22)_ = 6.42, *p* = 0.006; α4α6*, F_(2,21)_ = 0.89, *p* = 0.424). Similar to previous investigations [10], nicotine exposure produced a 157%, 154%, and 184% increase in nAChR density for α4*, α6*, and α4α6* nAChRs (Fig. 5C, not significant). In mice exposed to nicotine plus WS3/WS23, we observed an increase in α4* and α6* nAChR density compared to PGVG (*p* < 0.001) and 0.01, respectively (Fig. 5C, Table S12). Additionally, α4* nAChRs exhibited a significant increase different from nicotine alone (*p* < 0.05, Fig. 5C).

## Discussion

### Like menthol, synthetic coolants are not an inert ‘characterizing’ flavor

Electronic nicotine delivery systems (ENDS) continue to evolve in response to regulatory actions targeting menthol, leading to the emergence of synthetic cooling agents such as WS-3 and WS-23 as menthol substitutes. Despite their use, little is known about how coolants affect the abuse liability of nicotine. Here, we show that WS-3 combined with WS-23 significantly enhance nicotine reinforcement-related behavior and dopamine signaling in the NAc core, effects that are comparable in magnitude to menthol. Furthermore, combination of WS-3 and WS-23 enhance both cue-evoked and nicotine-evoked dopamine signaling in the NAc core. While additional experiments are needed to determine the precise mechanism, this enhancement may involve increased intrinsic excitability of VTA dopamine neurons and potentiation of nicotine-induced α4* and α6* nAChR upregulation. Together, these findings identify synthetic cooling agents as pharmacologically active modulators of nicotine reinforcement rather than inert sensory additives.

### Synthetic coolants amplify NAc core dopamine neurotransmission

Consistent with the behavioral findings, fiber photometry recordings of dLight1.3b fluorescence revealed that WS-3/WS-23 enhanced nicotine-evoked dopamine signals in the NAc core. Both cue-evoked and eVape-triggered responses were significantly elevated in the synthetic coolant group relative to nicotine alone. These dynamic changes in dopamine signaling reflect increased salience of both conditioned and unconditioned stimuli associated with nicotine intake and mirror the reinforcement-enhancing properties previously attributed to menthol [9, 10, 14, 15, 30].

Complementary FSCV experiments confirmed that WS-3/WS-23 exposure increased electrically-evoked (tonic and phasic) dopamine release in the NAc core. We and others have shown that menthol enhances NAc core dopamine release significantly more than nicotine alone [8, 12]. Here we observed a similar result but also observed that WS-3/WS-23 elicited similar effects to menthol. Because phasic dopamine signaling is critical for encoding reward prediction and driving reinforcement learning, this enhancement provides additional evidence to explain the impact of coolants on nicotine reinforcement-related behavior.

### Synthetic coolants enhance VTA dopamine neuronal intrinsic excitability and nAChR upregulation

Whole-cell patch-clamp electrophysiology targeting VTA dopamine neurons revealed WS-3/WS-23 exposure with nicotine significantly increased neuronal intrinsic excitability beyond that of nicotine alone. Specifically, these synthetic coolants reduced rheobase and elevated maximal firing frequency, mirroring previous findings with menthol [10]. Both nicotine and nicotine plus WS-3/WS-23 enhanced VTA dopamine neuron excitability in response to acute applications of 500 nM nicotine. Several investigations have characterized nicotine-induced enhancement of VTA dopamine neurons partly due to suppression of GABA-mediated inhibitory tone [43–45]. Given that we observed all nicotine-treated conditions (nicotine-alone or nicotine plus WS3/WS23), this may be involved with the enhancements in nicotine-stimulated VTA dopamine release. However, we observed that the combination of nicotine and WS-3/WS-23 did not produce an enhancement over nicotine-alone in comparison to our previous findings with menthol [10].

A key distinction between menthol and WS-3/WS-23 may involve their effects on α4α6* nAChRs. Previous work demonstrated that menthol enhances nicotine-induced upregulation of α4α6* nAChRs [10]. In the present study, WS-3/WS-23 significantly enhanced α4* and α6* nAChR upregulation; but we did not detect a significant effect on α4α6* nAChR density. Notably, the α4α6* measurements exhibited substantial variability and the overall analysis was not significant, limiting our ability to determine whether nicotine-alone and nicotine plus WS-3/WS-23 differentially upregulate these nAChRs. Because acute responses to smoking-relevant concentrations of nicotine (≤500 nM) have been linked to α4α6* nAChRs [10, 34, 46, 47], one possibility is that differences in α4α6* nAChR upregulation contribute to the lack of enhanced nicotine-stimulated excitability observed following WS-3/WS-23 exposure. However, additional studies will be necessary to test this.

Together, these results provide converging behavioral, neurochemical, and electrophysiological evidence that coolants enhance nicotine’s reinforcing properties. At present, these results are descriptive and fail to identify specific causal mechanisms. However, potentiation of NAc core dopamine signaling, changes in nAChR upregulation, and alterations in neuronal intrinsic excitability provide prospective areas for designing mechanistic studies to determine causal factors. These effects we observed are comparable to those of menthol, a chemical flavor already recognized for its role in promoting nicotine dependence and now subject to increasing regulatory scrutiny. Given that WS-3 and WS-23 are currently unregulated in ENDS products, their widespread use represents an emerging risk factor for nicotine dependence. From a translational perspective, these findings underscore the need for regulatory agencies to evaluate the abuse liability of synthetic coolant additives currently marketed as inert alternatives to menthol.

## Supplementary information

Supplemental Material

## Data Availability

Experimental data are available by request to BJH.
