# Supplementary material for: Synthetic cooling agents enhance nicotine reinforcement and mesolimbic dopamine signaling
Source: Neuropsychopharmacology. 2026 Jul 23;51(10):1748–57. doi: 10.1038/s41386-026-02485-0 (PMC13486633; doi:10.1038/s41386-026-02485-0)
Supplement: Supplementary file 1 [file 41386_2026_2485_MOESM1_ESM.docx]

**Supplemental Data**

**Figure S1.** Mean active and inactive nose pokes for male and female mice assigned to PGVG (A, E), 6 mg/mL nicotine (B, F), 6 mg/mL nicotine plus 15 mg/mL menthol (C, G), and 6 mg/mL nicotine plus WS-3/WS-23 (D, H). Data are mean ± SEM. These data are from the same mice presented in Figure 1 of the main paper.

**Figure S2.** EVSA and dLight 1.3b recordings from mice assigned only WS-3/WS-23. (A, F) Active and inactive nose pokes for male (A) and female (F) mice assigned only to WS-3/WS-23. (B, G) Earned eVape deliveries for male and female mice. (C, D, H, I) Representative wave forms for cue-stimulated and eVape-stimulated dLight 1.3b fluorescence for mice assigned to FR3 with WS-3/WS-23 only. (E, J) Mean (with SEM) peak dLight 1.3b fluorescence of male (n = 4) and female (n = 4) mice exposed to WS-3/WS-23 only. All dLight 1.3b fluorescence recordings are from NAc core.

| Table S1 | | | | |
| --- | --- | --- | --- | --- |
| Male EVSA active nose pokes (Fig 1B) | | | Male EVSA earned eVape deliveries (Fig 1C) | |
| **Fixed effects** | ***F*** | ***p* value** | ***F*** | ***p* value** |
| Session | F_(1.8, 40)_ = 12.99 | <0.0001 | F_(1.8, 40)_ = 6.280 | 0.0018 |
| e-liquid | F_(3, 22)_ = 6.722 | 0.0022 | F_(3, 22)_ = 7.373 | 0.0014 |
| session x e-liquid | F_(5.5, 40)_ = 3.622 | 0.0070 | F_(5.5, 40)_ = 3.23 | 0.0052 |
| Two-Way ANOVA (Mixed Effects) with Geisser-Greenhouse Correction | | | | |

| Table S2. Male FR1, FR2, and FR3 (Fig 1D, 1E, 1F) | | |
| --- | --- | --- |
| **FR** | ***F*** | ***p* value** |
| FR1 | F_(3,22)_ = 5.609 | 0.0052 |
| FR2 | F_(3,22)_ = 9.212 | 0.0004 |
| FR3 | F_(3,22)_ = 5.395 | 0.0006 |
| One-Way ANOVA | | |

| Table S3 | | | | |
| --- | --- | --- | --- | --- |
| Female EVSA active nose pokes (Fig 1G) | | | Female EVSA earned eVape deliveries (Fig 1H) | |
| **Fixed effects** | ***F*** | ***p* value** | ***F*** | ***p* value** |
| Session | F_(3.8, 90.9)_ = 28.45 | <0.0001 | F_(3.421, 82.1)_ = 3.713 | 0.0113 |
| e-liquid | F_(3, 24)_ = 76.67 | <0.0001 | F_(3, 24)_ = 28.23 | <0.0001 |
| session x e-liquid | F_(11.36, 90.9)_ = 7.186 | <0.0001 | F_(10.26, 82.1)_ = 1.805 | 0.0707 |
| Two-Way ANOVA (Mixed Effects) with Geisser-Greenhouse Correction | | | | |

| Table S4. Female FR1, FR2, and FR3 (Fig 1I, 1J, 1K) | | |
| --- | --- | --- |
| **FR** | ***F*** | ***p* value** |
| FR1 | F_(3,24)_ = 12.02 | <0.0001 |
| FR2 | F_(3,24)_ = 35.66 | <0.0001 |
| FR3 | F_(3,24)_ = 7.34 | 0.0013 |
| One-Way ANOVA | | |

| Table S5. eVape Self-administration Comparison by sex | | |
| --- | --- | --- |
| **FR** | ***F*** | ***p* value** |
| Interaction | F_(3,46)_ = 0.116 | 0.950 |
| Sex | F_(1,46)_ = 0.816 | 0.3711 |
| e-liquid | F_(3,46)_ = 12.51 | <0.0001 |
| Two-Way ANOVA | | |

| Table S6. Photometry (Fig 2E, 2J) | | |
| --- | --- | --- |
| **Fixed effect** | ***F*** | ***p* value** |
| Cue (Fig. 2E) | F_(3,31)_ = 10.16 | <0.0001 |
| eVape (Fig. 2J) | F_(3,31)_ = 47.63 | <0.0001 |
| One-Way ANOVA | | |

| Table S7. FSCV (Fig 3F) | | |
| --- | --- | --- |
| **Fixed effects** | ***F*** | ***p* value** |
| Interaction | F_(3,50)_ = 0.4447 | 0.7221 |
| Stim Frequency | F_(1,50)_ = 1.323 | 0.2555 |
| e-liquid | F_(3,50)_ = 46.02 | <0.0001 |
| Two-Way ANOVA (Repeated Measures) with Geisser-Greenhouse Correction | | |

| Table S8. Rheobase (Fig 4C) | | |
| --- | --- | --- |
| **Fixed effect** | ***F*** | ***p* value** |
| Rheobase (Fig. 4C) | F_(2,17)_ = 21.62 | <0.0001 |
| One-Way ANOVA | | |

| Table S9. IV Plot (Fig 4D) | | |
| --- | --- | --- |
| **Fixed effects** | ***F*** | ***p* value** |
| Interaction | F_(3,19)_ = 2.034 | 0.1390 |
| Current | F_(2,19)_ = 9.18 | 0.0025 |
| e-liquid | F_(2,11)_ = 5.912 | 0.018 |
| Current Step | F_(11,242)_ = 29.05 | <0.0001 |
| Two-Way ANOVA | | |

| Table S10. Nicotine Application VTA Dopamine neurons (Fig 4F) | | |
| --- | --- | --- |
| **Fixed effects** | ***F*** | ***p* value** |
| Interaction | F_(2,16)_ = 10.6 | 0.0012 |
| e-liquid | F_(2,16)_ = 4.080 | 0.0370 |
| Acute Nicotine | F_(1,16)_ = 31.41 | <0.0001 |
| Mouse | F_(16,16)_ = 2.331 | 0.0502 |
| Two-Way ANOVA | | |

| Table S11. Nicotine Application VTA GABA neurons (Fig 4F) | | |
| --- | --- | --- |
| **Fixed effects** | ***F*** | ***p* value** |
| Interaction | F_(2,9)_ = 17.9 | 0.0007 |
| e-liquid | F_(2,9)_ = 7.69 | 0.0113 |
| Acute Nicotine | F_(1,9)_ = 44.4 | <0.0001 |
| Mouse | F_(9,9)_ = 1.39 | 0.316 |
| Two-Way ANOVA | | |

| Table S12. Microscopy (Fig 5C) | | |
| --- | --- | --- |
| **Fixed effect** | ***F*** | ***p* value** |
| α4-mCherry | F_(2,22)_ = 10.46 | 0.0006 |
| α6-GFP | F_(2,22)_ = 6.419 | 0.0064 |
| α4α6* | F_(2,21)_ = 0.8931 | 0.4244 |
| One-Way ANOVA | | |
